# Characterization of mice with a deletion of protein kinase G type I in cardiomyocytes and the effect on cardioprotection through either postconditioning or mitochondria-targeted *S*-nitrosothiol

**DOI:** 10.1186/1471-2210-11-S1-O15

**Published:** 2011-08-01

**Authors:** Carmen Methner, Robert Lukowski, Franz Hofmann, Michael Murphy, Thomas Krieg

**Affiliations:** 1 Clinical Pharmacology Unit, University of Cambridge, UK; 2 Pharmakologie, klinische Pharmazie und Toxikologie, Universität Tübingen, Germany; 3 Forschergruppe 923, Technische Universität München, Germany; 4 MRC Mitochondrial Biology Unit, Cambridge, UK

## Background

Protein kinase G type I (PKGI/cGMP kinase I) plays a critical role in survival signalling of pre- and postconditioning. However, it is unclear whether cGKI exerts its protective effects in the cardiomyocyte or if other cardiac cell types are involved, and whether nitric oxide (NO) has cGKI-independent effects on cardiomyocytes mitochondria.

## Objective

We developed mice with a cardiomyocyte-specific ablation of the cGKI gene (CMG-KO) and tested whether protection against reperfusion injury by ischemic postconditioning (IPost), soluble guanylyl cyclase (sGC) activation, the adenosine A_2B_ receptor (A_2B_AR), or the mitochondria-targeted *S*-nitrosothiol (MitoSNO) was affected. MitoSNO accumulates within mitochondria, driven by the membrane potential, where it generates NO^•^ and *S*-nitrosated thiol proteins [1].

## Methods and results

**Figure 1:**
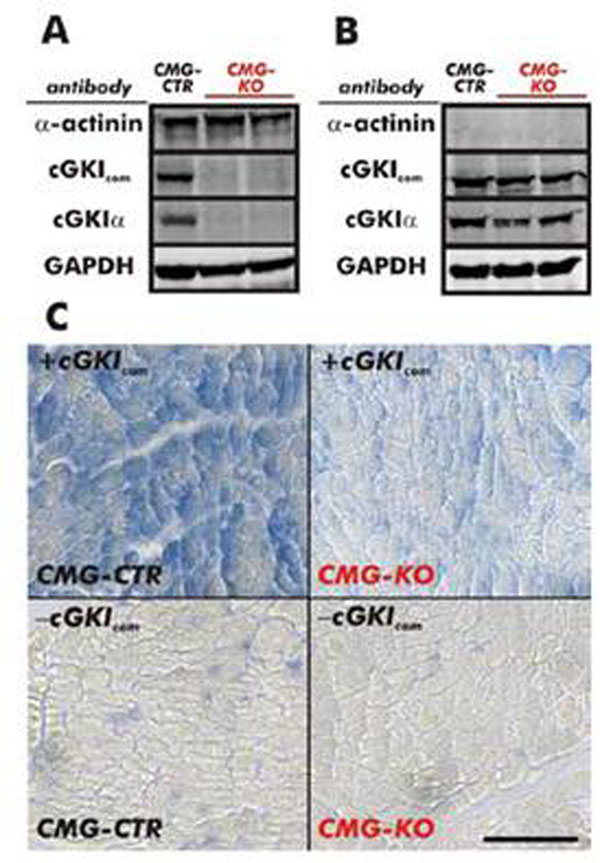
Representative Western Blots of **A** left ventricle and **B** cerebellum of CMG-KO and control mice from the same litters on a C57/Bl6N genetic background (CMG-CTR), clearly indicating the specific absence of cGKI in the heart. **C** Immunohistochemistry of cardiac tissue.

Conditional mice with floxed cGKI alleles were crossed to the MLC2a-Cre transgenic mice. Western Blot and immunohistochemistry confirmed that the Cre-mediated recombination produced the cGKI knock-out specifically in atrial and ventricular cardiomyocytes but not in other organs (Figure 1).

*In situ* hearts of underwent 30 min of regional ischemia followed by 2 h of reperfusion. As expected, in the control animals all interventions at early reperfusion lead to profound infarct size reduction: IPost (six cycles of 10 sec reperfusion and 10 sec of coronary occlusion), treatment with the specific sGC activator BAY 58-2667 (BAY58), the selective A_2B_AR agonist BAY 60-6583 (BAY60), as well as MitoSNO. In contrast, the hearts of CMG-KO animals were not protected by BAY58, whereas the protective effects of IPost, BAY60, and MitoSNO were unaffected by the lack of cGKI.

**Figure 2:**
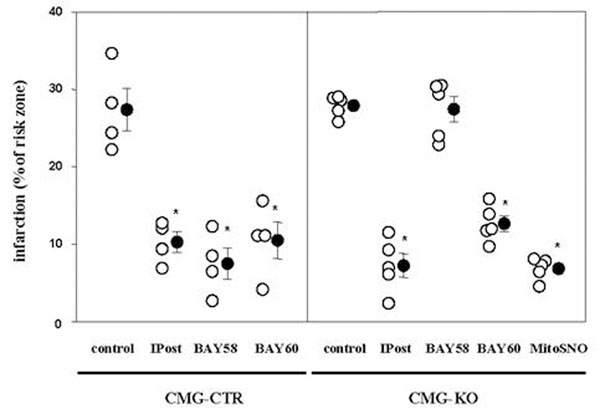
Results of open chest experiments. As expected, either IPost, activation of sGC with BAY58 or A_2B_AR activation with BAY60 resulted in a profound decrease of infarct size in CMG-CTR. While BAY58 failed to protect in the CMG-KO animals, IPost, BAY60, and MitoSNO still afforded protection, suggesting a signaling independent on cGKI in the cardiomyocyte. *p<0.05 vs. control.

## Conclusion

While cardiomyocyte cGKI is important for the protection afforded via cGMP-signalling, beneficial effects of IPost, activation of the A_2B_AR, as well as direct NO effects via mitochondrial *S*-nitrosylation does not depend on cGKI in cardiomyocytes.
